# ADRD Caregiving Experiences and Health by Race, Ethnicity and Care Recipient Geographic Context

**DOI:** 10.1093/geroni/igab046.3557

**Published:** 2021-12-17

**Authors:** Tiffany Kindratt, Dominigue Sylvers, Aya Yoshikawa, Mónika López Anuarbe, Noah Webster, Erin Bouldin

**Affiliations:** 1 University of Texas at Arlington, Arlington, Texas, United States; 2 University of Michigan School of Public Health, University of Michigan Institute for Social Resarch, Michigan, United States; 3 Texas Woman's University, Texas Woman's University, Texas, United States; 4 Connecticut College, New London, Connecticut, United States; 5 University of Michigan, Ann Arbor, Michigan, United States; 6 Appalachian State University, Boone, North Carolina, United States

## Abstract

Few studies have examined how the intersectionality of geographic context and race/ethnicity influences Alzheimer’s disease and related dementia (ADRD) caregiving. Our aims were to determine whether 1) caregiver experiences and health differed across urban and rural areas; and 2) these links were moderated by caregiver race/ethnicity. We used data from the 2017 National Health and Aging Trends Study and National Study of Caregiving. The sample included caregivers (n=808) of care recipients ages 65+ with ‘probable’ ADRD (n=482). Geographic context was defined as care recipient’s residence in metro (urban) or non-metro (rural) counties. Outcomes included caregiving experiences (burden, gains, life impacts, service/resource use) and health (self-rated, anxiety, depression symptoms, chronic health conditions). Bivariate analyses indicated that non-metro ADRD caregivers were less racially/ethnically diverse (82.7% white) and more were spouses/partners (20.2%). Among racial/ethnic minority ADRD caregivers, non-metro context was associated with having more chronic conditions (p<.01), providing less care (p<.01), and not co-residing with care recipients (p<.001). Amid white ADRD caregivers, non-metro context was associated with not reporting caregiving was more than they could handle (p<.05) and finding financial assistance for caregiving (p<.05). Multivariate regression analyses demonstrated that non-metro minority ADRD caregivers had 3.09 times higher odds (95% CI=1,02-9.36) of reporting anxiety in comparison to metro minority ADRD caregivers. Geographic context shapes ADRD caregiving experiences and caregiver health differently across racial/ethnic groups. Despite higher rates of ADRD and ADRD-related mortality in non-metro areas, findings suggest both positive and negative aspects of caregiving among White, Black, and Hispanic ADRD caregivers.

